# Editorial: Perspectives in fungal pathogenesis: 2025

**DOI:** 10.3389/fcimb.2026.2020473

**Published:** 2026-10-26

**Authors:** Joseph T. Nickels, Yuanwei Zhang, Scott E. Gygax, Daniel Henk

**Affiliations:** 1 Genesis Biotechnology Group, Hamilton, NJ, United States; 2 Rutgers Center for Lipid Research, New Jersey Institute for Food, Nutrition, and Health, Rutgers University, New Brunswick, NJ, United States; 3 Nanjing Normal University, College of Life Sciences, Nanjing, Jiangsu, China; 4 Thomas Jefferson University, Philadelphia, PA, United States; 5 University of Bath, Bath, United Kingdom

**Keywords:** fungi, host, inflammation, pathogenesis, taxonomy

Pathogenic microbes exploit both host and microbial systems to cause disease, hijacking host pathways to enhance their virulence (Strickland and Shi, 2021; Konig et al., 2021). Virulence thus arises from the coordinated activity of complex molecular networks in both the host and the microbe (Mendes-Giannini et al., 2005; Camilli et al., 2021). Although *Candida albicans* and *Nakaseomyces glabratus* (formerly *Candida glabrata*) are among the most common fungal pathogens in immunocompromised individuals (Katsipoulaki et al., 2024), other fungal species are increasingly emerging as drivers of disease progression (Wiederhold, 2021).

This series brings together six articles that expand our understanding of newly recognized pathogenic fungi and the mechanisms by which they contribute to disease progression. Collectively, these studies are intended to advance knowledge of the fundamental pathogenic processes through which diverse fungal species increase disease burden, morbidity, and mortality in affected individuals.

Sporotrichosis is a fungal disease that can occur in immunocompromised individuals (Orofino-Costa et al., 2017). Cases typically present as lymphocutaneous lesions that may progress to open sores or ulcers. When disseminated, the infection can spread to the lungs, bones, and central nervous system, becoming life-threatening. Yuan et al., 2026 provide a comprehensive review of the chronological evolution of the literature based on the increasing understanding of sporotrichosis, an infectious disease caused by the fungus *Sporothrix schenckii.* They identified the need for more definitive research on the mechanisms underlying disease progression, as well as investigation into therapeutic drug discovery. The authors emphasized that collaborative efforts could accelerate progress to the development of effective treatments for immunocompromised individuals affected by this deadly disease.

Individuals with autoimmune rheumatic diseases are susceptible to microbial infections (Caminer et al., 2017). Alterations in the host microbiota have been associated with autoimmune disorders. Zhang et al., 2025 show that the fungus *Kazachstania pintolopesii*, isolated from a monkey model of ankylosing spondylitis, induces PANoptosome assembly and activity, promoting immune cell death through apoptosis, pyroptosis, and necroptosis (Sun et al., 2024); however, the molecular mechanisms underlying this effect remain unclear. They demonstrate that *K. pintolopesii* disrupts immune homeostasis and causes joint disease by activating an immune-endothelial-fibroblast cascade that drives inflammatory cytokine production and fibrosis. Bioinformatic analyses further suggest T-cell activation in osteoblasts. These findings identify *K. pintolopesii* as a contributor to autoimmune disease and open new avenues for therapeutic strategies, with future mechanistic studies needed to define how this fungus activates PANoptosis at the molecular level.

*Candida albicans* and *Nakaseomyces glabratus* represent a major health concern causing morbidity and mortality in immunocompromised individuals, a challenge intensified by the emergence of antifungal-resistant strains (Katsipoulaki et al., 2024). Increased infections caused by emerging *Candida* species, including *C. krusei*, *C. parapsilosis*, *C. tropicalis*, and *C. auris*, have been reported in neonatal units, nursing homes for older adults, and patients with cancer (Barantsevich and Barantsevich, 2022). Fernandes et al., 2025 review recent literature on *Candida* spp., with emphasis on the taxonomic reclassification of these and other clinically relevant fungi, their virulence factors, pathogenic mechanisms, and molecular basis of antifungal resistance. They summarize topics such as biofilm formation and its contribution to antifungal resistance and the role of adhesins in host-pathogen interactions. The review also examines the biology of *Candida* species and their resistance to the major classes of antifungal agents, including azoles, echinocandins, and polyenes.

Several fungal species can infect a wide range of plants (Sun et al., 2020). Singh et al., 2025 expand the taxonomic nomenclature of cercosporoid hyphomycetous fungi by proposing two new genera within the family *Mycosphaerellaceae*. Their classification is supported by molecular analyses linked to fungal diversity and pathogenic potential. Using multiple approaches, the authors identified these isolates as novel cercosporoid fungi, providing a refined framework for understanding their taxonomy and evolutionary relationships. The two newly proposed genera are *Marcstadlera*, represented by *M. malloti*, and *Neoclypeosphaerella*, represented by *N. calotropidis*. These two species differ in conidial loci and hila morphology. These findings broaden our understanding of pathogenic fungi associated with severe plant diseases.

Pulmonary aspergillosis is caused by fungal pathogenic *Aspergillus* species and occurs predominately in individuals with underlying risk factors or impaired immunity (Machado et al., 2024). This disease primarily affects immunocompromised individuals and pediatric patients. Accurate diagnosis remains challenging because pulmonary aspergillosis can be difficult to distinguish from non-aspergillosis respiratory infections due to overlapping clinical symptoms, difficulties in obtaining appropriate respiratory specimens, and limited availability of diagnostic fungal biomarker tests. In their study, Yang et al., 2025 identified independent risk factors that may help distinguish pediatric patients with pulmonary aspergillosis from those without this fungal infection, including viral coinfection, absence of fever, and elevated serum β-D-glucan levels. Thus, the authors construct a diagnostic framework that will help physicians diagnose and treat aspergillosis.

The infectivity-related kinases Irk2 and Irk5 are important virulence determinants in *Cryptococcus neoformans* infections (Lee, 2016). They support the ability of *C. neoformans* to adapt to metabolic environments, making them targets for drug discovery (Iyer et al., 2021). Ma et al., 2025 used integrated proteomic and metabolomic approaches to define the metabolic changes associated with deletion of *Irk2* or *Irk5*. Proteomic KEGG analysis linked these kinases to pathways regulating carbohydrate, amino acid, and lipid metabolism. Metabolomic profiling implicated them in aminoacyl-tRNA and amino acid biosynthesis. A potential role for Irk2 and Irk5 in mitochondrial function was uncovered, noting increased ATP levels in the single-gene deletion strains. These findings suggest the reduced virulence of the *irk2*Δ and *irk5*Δ mutants may result from altered mitochondrial oxidative phosphorylation and disrupted ATP homeostasis.

In summary, this series emphasizes the dynamic and multifaceted interactions between fungal pathogens and their hosts. The articles examine fungal disease biology, including host immune activation, pathogen-driven inflammatory responses, fungal virulence mechanisms, metabolic adaptation, and mitochondrial function. They highlight the complexity of pathogenesis and identify gaps and therapeutic opportunities. Integrating insights from clinical observations, experimental models, and systems-level analyses, the collection provides a foundation for research aimed at improving the diagnosis, treatment, and prevention of pathogenic fungal diseases.
